# A Critical Appraisal of the Links Between Video Gaming, Lifestyle Factors, Diet and Eating Behaviour: A Narrative Review

**DOI:** 10.3390/nu18060967

**Published:** 2026-03-19

**Authors:** Svetlana Deric, Thanaporn Kaewpradup, Sirichai Adisakwattana, Ellise Stirling, Blossom Stephan, Van Nguyen, Leticia Radin Pereira, Hannah Velure Uren, Mario Siervo

**Affiliations:** 1 Faculty of Health Sciences, School of Population Health, Curtin University, Perth, WA 6102, Australia; svetlana.deric@postgrad.curtin.edu.au (S.D.); ellise.stirling@curtin.edu.au (E.S.); van.h.nguyen@curtin.edu.au (V.N.); hannah.uren@curtin.edu.au (H.V.U.); 2 Center of Excellence in Phytochemical and Functional Food for Clinical Nutrition, Department of Nutrition and Dietetics, Faculty of Allied Health Science, Chulalongkorn University, Bangkok 10330, Thailand; thanaporn.k@chula.ac.th (T.K.); sirichai.a@chula.ac.th (S.A.); 3 Curtin-Chulalongkorn Collaborative Centre for Nutrition and Food Research and Education, Curtin University, Perth, WA 6845, Australia; 4 Curtin Dementia Centre of Excellence, Enable Institute, Curtin University, Perth, WA 6102, Australia; blossom.stephan@curtin.edu.au; 5 Department of Community Health Sciences, Cumming School of Medicine, University of Calgary, 3280 Hospital Drive NW, Calgary, AB T2N 4Z6, Canada; leticia.radinpereira@ucalgary.ca; 6 Curtin Medical Research Institute (CMRI), Curtin University, Perth, WA 6102, Australia

**Keywords:** video gaming, diet, physical activity, eating behaviour, sleep

## Abstract

**Background:** Video gaming is a highly prevalent leisure activity globally, with complex associations across multiple health domains. **Methods:** This narrative review critically appraised the existing literature identified through targeted searches of PubMed and Google Scholar to synthesise evidence on associations between video gaming and psychosocial stress, physical activity, sleep quality, eating behaviour, and diet quality. Theoretical, biological, and psychosocial mechanisms underlying these relationships were examined, and methodological limitations and research gaps were identified. **Results:** The relationships between video gaming and health outcomes appear bidirectional and context dependent. While video gaming may provide short-term stress relief and social connection, frequent or prolonged gaming may be associated with sedentary behaviour, physical inactivity, impaired sleep quality, disrupted eating patterns, and poorer diet quality. These associations may vary by age, sex, gaming duration, timing, content, and motivational drivers. Gaming-related cognitive absorption and physiological arousal may influence appetite regulation, sleep onset, and stress responses, while temporal displacement and environmental factors, such as food availability and marketing exposure, also contribute. **Conclusions:** An integrated biopsychosocial framework is proposed to describe the interconnected pathways through which video gaming may influence health, incorporating biological arousal, psychological immersion, and social and environmental contexts. Significant gaps remain, including the scarcity of longitudinal studies, limited consideration of moderating factors, and inconsistent measurement of gaming behaviours. Addressing these gaps is essential for refining public health surveillance and supporting the development of evidence-based strategies that promote healthy gaming behaviours while preserving potential psychosocial benefits.

## 1. Introduction

The video game industry has seen a significant transformation over recent decades, evolving from arcade-based entertainment to sophisticated, immersive experiences facilitated by technological advancement and internet connectivity [1]. Video gaming is a global leisure activity, with an estimated 3.3 billion players worldwide as of 2024 [2], and prevalence is highest among younger age groups, with over 90% of adolescents and young adults reporting regular gaming compared to lower frequencies later in life [3]. While males continue to game more frequently and for longer durations, females now represent approximately half of the global gaming population [4]. An estimated 92% of Australian households engage in video gaming [5], and while the gender gap still persists with males engaging more frequently and for longer durations, female participation has increased substantially and represents more than 40% of the video gaming population [5]. The age distribution of gaming populations has also seen some demographic changes, with current data indicating that 68% of Australian gamers fall within 18–64 years, reflecting the increased prevalence of video gaming across all life stages from childhood through late adulthood [5].

The types of games played and the motivations driving gaming engagement also differ substantially across the lifespan. Children and adolescents predominantly engage with action, adventure, role-playing, and competitive multiplayer games, with entertainment and peer-based social interaction serving as primary motivators [6]. Young adults represent the most active gaming demographic and tend to engage across a broad range of genres, including first-person shooters, multiplayer online games, and sports simulations, often driven by competition, achievement, and social belonging [6]. In contrast, older adults are more likely to engage with puzzle, strategy, casual, and cognitive training games, with motivations centred on cognitive stimulation, relaxation, and social connection rather than competition [7]. These age-related differences in gaming preferences and motivational profiles may have important implications for health outcomes. For example, competitive and immersive gaming in younger subjects may amplify physiological arousal and social pressure [8], whereas cognitive gaming in older adults may confer protective benefits for mental health and cognitive function [9]. Understanding how psychosocial factors interact with gaming type and age is therefore essential to interpret the health-related evidence presented in this review.

The links between video gaming and health outcomes could be conceptualised using multiple theoretical frameworks [10,11]. The displacement hypothesis suggests that time spent video gaming may replace time that could otherwise be dedicated to health-promoting activities such as physical exercise, meal preparation, or adequate sleep [10,12]. This perspective may explain the observed negative correlations between video gaming duration and various health outcomes across different age groups. However, while traditional sedentary gaming may displace health-promoting activities such as outdoor play and exercise, this relationship is not universally applicable. Emerging active gaming technologies and exergames could promote physical activity and movement and be part of lifestyle interventions or behavioural strategies to develop personalised approaches to encourage physical movement [13,14,15].

The motivational factors driving gaming behaviour may be linked to the fulfilment of specific psychosocial needs [16]. These motivational drivers have been shown to have age-related variation, with entertainment and social interaction with peers being more frequent among younger cohorts, whereas cognitive maintenance, social engagement and promotion of physical health could be more relevant in older adult populations [17,18]. This psycho-social interpretation of video gaming trends could explain why video gaming persists despite potential health consequences, as the immediate psychological benefits may outweigh or obscure longer-term health risks. Bio-psychosocial models of the potential factors integrate physiological (autonomic arousal, sleep disruption), psychological (stress response, cognitive absorption), and social (gaming culture, peer influences) mechanisms [19]. The model suggests a complex bidirectional relationship between video gaming and health outcomes, which may operate differently across life stage periods. A significant gap currently exists in the understanding of the influence of video gaming on physical and mental health domains across the lifespan.

From a public health perspective, video gaming represents a unique behavioural exposure that is both highly prevalent and deeply embedded in daily routines, particularly among younger groups [20]. Unlike other sedentary behaviours, gaming often involves prolonged cognitive engagement, social interaction, and physiological arousal, suggesting that its health effects may not be fully captured by traditional screen-time metrics [11]. Importantly, gaming behaviours are increasingly shaped by platform design, monetization strategies, and online social environments, which may amplify or mitigate health impacts across the life course [11,21].

For the purposes of this review, the term “gamer” is used broadly to refer to individuals who regularly engage with video games across any platform, including consoles, personal computers, mobile devices, and handheld systems. We acknowledge that the field currently lacks a universally accepted definition, and that studies operationalise gaming engagement in varied ways, ranging from simple frequency or duration thresholds (e.g., ≥1 h per day, ≥7 h per week) [22] to standardised instruments such as the Internet Gaming Disorder Scale or the Gaming Disorder Scale [23]. For clarity, this review distinguishes, where evidence permits, between casual or recreational gaming (infrequent, low-intensity engagement), regular or frequent gaming (consistent engagement as a primary leisure activity), and problematic or disordered gaming (compulsive patterns associated with functional impairment). Different types of gaming, including sedentary gaming, active or exergaming, mobile gaming, and augmented reality gaming, are also discussed. Therefore, the aim of this narrative review is to synthesise current evidence on associations between video gaming and key health-related domains (psychosocial stress, physical activity, sleep quality, eating behaviours, and diet quality) across the lifespan. In addition, we propose a biopsychosocial framework to describe potential mechanisms (e.g., temporal displacement, attentional allocation, autonomic arousal, and environmental influences) and to generate testable hypotheses to direct future research.

## 2. Materials and Methods

Relevant literature was identified through the authors’ expertise and targeted searches of the electronic databases PubMed and Google Scholar, conducted between December 2025 and February 2026, with a focus on publications from 2000 to 2025. The following search terms were used, individually and in combination: video gaming, exergames, active video games, diet quality, eating behaviour, physical activity, sleep, psychosocial stress, food intake, and diet. Searches were conducted in English only. Inclusion priority was given to peer-reviewed experimental studies, longitudinal studies, and cross-sectional surveys with validated outcome measures. Systematic reviews and meta-analyses relevant to the health domains were also examined. Grey literature, conference abstracts, and non-peer-reviewed sources were excluded. A summary of key studies that have investigated the link between video gaming and health outcomes across the life course is provided in Table 1.

## 3. Results

### 3.1. Video Gaming and Psychosocial Stress

Evidence from several studies suggests a bidirectional relationship between video gaming and psychosocial stress, with video-gaming demonstrating the stress-relieving properties [56]. Prior research demonstrated that more than half of participants who engaged in immersive online gaming reported mood improvement and stress reduction [29]. Psychological absorption and dissociation have been identified as key mechanisms, and deeply immersive games may redirect attention away from existing life stressors and facilitate a relaxation response [57]. Participants experiencing greater work or academic strain were shown to more frequently engage in video gaming as a coping strategy compared to less-stressed participants [24]. However, this stress-relief function could be context-dependent, such as the presence of violence in the video gaming experience or increased levels of competitiveness in video game online communities [58,59]. A pilot clinical trial compared physiological responses to video gaming with violent content versus television viewing and found significantly higher cortisol levels and blood pressure responses during gaming sessions [32].

Video gaming can also serve as a maladaptive coping mechanism among individuals with high-frequency usage patterns. Compensatory escapism, characterised by avoidance-focused coping behaviours, provides immediate stress alleviation, but ultimately intensifies stress through neglect of underlying issues [60]. University students with higher perceived stress have been shown to be at increased risk of developing problematic gaming patterns [61], and video gaming during exam periods was linked to poorer stress management and academic outcomes [46].

Importantly, individual differences appear to moderate the stress–gaming relationship. Differences in personality traits, coping styles, and vulnerability to problematic gaming may determine whether gaming functions as an adaptive or maladaptive strategy [62]. Individuals with high emotional regulation skills may benefit from short, controlled gaming sessions, whereas those with avoidant coping tendencies may be at increased risk of excessive use and escalating stress over time [63]. Developmental stage is also relevant, as adolescents may lack the cognitive maturity to self-regulate gaming duration and emotional investment, increasing susceptibility to stress dysregulation [64]. These findings reinforce the need to distinguish between recreational, socially embedded gaming and problematic, compulsive patterns when interpreting associations with psychosocial stress.

The relationship between video gaming and stress appears to be moderated by both game characteristics and social context. Competitive games have been shown to increase physiological stress markers compared to cooperative games [65], whereas online social gaming has been associated with greater social connectedness that may protect against academic stress [66]. The growing literature on cooperative gaming suggests that game-related teamwork rather than competition may enhance social support and positive emotions [19], which highlights the importance of considering not just video gaming duration but also specific game features and social dynamics when evaluating links between video gaming, psychosocial stress, and health outcomes.

### 3.2. Video Gaming and Physical Activity

Video gaming is often criticised for displacing time that could otherwise be spent on physical activity as well as for being associated with diminished muscle performance [67]. Most of video games are generally sedentary, involving minimal movement and low energy expenditure [68,69], in contrast to recommendations that adults should engage in 150–300 min of moderate-intensity exercise per week [70]. The inverse associations between video gaming duration and physical activity level are well-established, demonstrating that frequent male gamers report significantly fewer daily physical activity minutes, reduced exercise frequency, and shorter exercise duration compared to non-gamers, while controlling for other media use [25,44,49]. In addition to time displacement, previous studies have shown that university students who frequently engage in video gaming reported multiple psychological barriers to physical activity participation, including increased fatigue, reduced motivation, and diminished physical self-efficacy [44]. These findings suggest that gaming-related physical inactivity involves complex psychological pathways rather than time constraints alone.

The emergence of physically interactive video gaming has, however, complicated this narrative. Exergames and active video games requiring physical movement have demonstrated potential benefits for physical activity promotion [71]. Research has demonstrated that active video games can provide meaningful physical benefits. For example, an 8-week study with young adults demonstrated improved fitness compared to a control group, while another study found that the physical activity energy expenditure during active gaming was comparable to moderate exercise intensity levels such as brisk walking [41,72]. A recent meta-analysis of 15 systematic reviews showed that active video games significantly improved balance (both static and dynamic) and lower limb strength in older adults compared to control groups. However, there were no significant benefits for cardiovascular fitness, upper body strength, or knee extension strength [73].

The long-term sustainability of the benefits of exergames remains questionable, potentially due to declining novelty effects, insufficient variety in engaging game content, and progressively reduced intrinsic motivation over extended play periods [74,75]. Exergames were found to initially increase physical activity in young adults, but adherence declined significantly after 6 weeks, with participants reverting to more sedentary gaming options [76]. Additionally, the negative association between gaming duration and physical activity may be moderated by sex, with a stronger inverse association observed among male participants [77]. This may reflect different patterns of gaming engagement, with males being more likely to engage in extended gaming sessions. Moreover, gaming motivation may influence the relationship between gaming and physical activity as in achievement-oriented gamers show stronger negative associations than socially motivated gamers [78]. This may be potentially due to more intensive and prolonged gaming sessions focused on progression and completion rather than social interaction [11]. While exergames offer promising opportunities to increase physical movement, their effectiveness as sustained physical activity interventions remains uncertain. Behavioural economics suggests that intrinsic motivation and long-term adherence are critical determinants of success, yet many active gaming interventions rely heavily on novelty rather than habit formation [79]. Furthermore, active gaming may coexist with, rather than replace, sedentary gaming, resulting in limited net gains in daily energy expenditure [35]. Public health strategies should therefore view exergames as a complementary tool rather than a standalone solution, potentially integrating them within broader behavioural interventions that address motivation and environmental support for physical activity.

Emerging evidence on augmented reality (AR) gaming offers an additional avenue for physical activity promotion [80]. Studies examining Pokémon GO, the most widely studied AR game to date, have reported short-term increases in daily step counts and light-to-moderate intensity physical activity among players compared to non-players [14,81]. However, these benefits appear to be largely transient, with engagement and physical activity gains diminishing substantially within weeks to months of initial uptake, consistent with novelty-driven motivation patterns observed in other exergame research [81]. AR gaming also differs fundamentally from traditional exergames as physical activity may be incidental to gameplay rather than a designed outcome, which may limit its public health utility as a deliberate intervention tool [15,82]. However, AR gaming may represent a low-barrier entry point for physical activity among individuals who would not otherwise engage in structured exercise, and its integration into broader lifestyle interventions warrants further investigation.

### 3.3. Video Gaming and Sleep Quality

The growing prevalence of video gaming as a pre-sleep activity has raised concerns regarding its impact on sleep quality. Sleep quality, which could be defined as satisfaction with sleep onset, duration, efficiency, and wakefulness [83], is particularly crucial for physical and brain health across the life course. Multiple physiological and psychological mechanisms may link gaming to sleep disruption. First, the blue light emitted from screens has been shown to suppress melatonin production and disrupt circadian rhythms [84]. This effect appears particularly pronounced for devices held closer to the face, such as handheld gaming systems and mobile phones [85]. Second, the cognitive and emotional arousal induced by gaming may interfere with the normal deactivation phase necessary for sleep onset [86].

Observational studies have consistently demonstrated associations between gaming and compromised sleep outcomes. For example, a cross-sectional study of 844 young adults found that gaming frequency significantly predicted later bedtimes, impaired sleep onset and duration, and increased daytime fatigue [34]. Similarly, university students engaging in gaming for more than 3 h daily demonstrated significantly poorer sleep quality assessed using the Pittsburgh Sleep Quality Index, with significant impairments in sleep onset latency and sleep efficiency [47,54]. A meta-analysis of 67 studies found that gaming had significant negative associations with sleep outcomes compared to other screen-based activities, such as television viewing [87]. The contrasting effects may reflect higher cognitive engagement associated with video gaming and the required alertness compared to passive media engagement [88]. The timing and context of video gaming may also impact sleep quality. Evening gaming seems to have a greater negative effect on sleep [88]. Each hour of video gaming after 8 pm was associated with a 28 min delay in sleep onset among university students, compared to evening studying or television viewing [38]. The social element of online gaming may be linked to sleep disruption, as multiplayer online games were associated with later bedtimes compared to single-player games [38]. Developmental considerations are particularly important when examining gaming-related sleep disruption. Adolescents and young adults exhibit a natural delay in circadian phase, which may be exacerbated by evening gaming and social gaming obligations that extend into late hours [88]. In contrast, older adults may experience heightened sensitivity to sleep fragmentation and autonomic arousal, potentially amplifying the adverse effects of gaming on sleep continuity [86]. These age-specific vulnerabilities suggest that uniform recommendations around gaming and sleep may be insufficient, and guidance based on life stage, gaming timing, and content is required.

The platform through which video gaming is accessed may have important implications for health outcomes, independent of gaming duration. Mobile and handheld gaming devices are held in closer proximity to the face than televisions or desktop monitors, which may amplify the suppressive effect of blue light on melatonin production and thereby increase the risk of sleep disruption [89]. Gaming in bed, a posture particularly associated with smartphone and tablet use, may further compound this risk by associating the sleep environment with arousal and cognitive stimulation, potentially disrupting conditioned sleep onset [90,91]. In contrast, console gaming on a television at a standard viewing distance may carry a somewhat lower blue light risk, though cognitive and emotional arousal effects remain [92].

Platform differences are also relevant to physical activity and eating behaviour. Mobile gaming, due to its portability, is more likely to occur during meals or in environments where snack food is readily accessible, potentially increasing the risk of distracted eating [93]. Console and PC gaming, more commonly conducted in dedicated gaming setups, may involve longer uninterrupted sessions and greater entrenchment in sedentary postures [69]. Age-related differences in platform use are also notable. Older adults and female gamers are disproportionately likely to engage via mobile devices, whereas young adult males predominantly use consoles and PCs [5,21]. These platform preferences may partly explain sex and age differences in the observed health associations, and future research should account for platform type as a meaningful moderating variable rather than treating all gaming as equivalent.

### 3.4. Video Gaming, Diet and Eating Behaviour

Video gaming has been suggested to disrupt eating behaviour via mechanisms of cognitive absorption and attentional allocation. Eating behaviour includes food-related practices related to food choice motivations, eating patterns, and eating-related psychosocial factors [94], which may be disrupted during video gaming. The cognitive absorption characteristic of immersive gaming can promote “mindless eating” by reducing attention allocated to food consumption [28]. For example, participants playing computer games during meals reported reduced fullness, poorer meal recall, and increased subsequent snacking compared to non-distracted eaters [95]. This attentional mechanism has been replicated across multiple studies, suggesting that frequent video game players reported significantly higher rates of distracted eating patterns, greater impairment in satiety recognition, and a higher caloric intake compared to non-gamers who engaged in television viewing and reading [50,96].

Video gaming also appears to disrupt normal eating and meal patterns. Longer gaming sessions may be associated with more frequent meal skipping and late-night eating, with participants reporting that they delay or skip meals to continue gaming sessions [40,54,97,98]. The timing of gaming-associated eating appears particularly problematic from a metabolic health perspective. Frequent video gaming later in the day may be linked to higher rates of night eating syndrome and altered glucose metabolism compared to daytime-only gamers [99]. The gaming environment is also often characterised by readily available, easily consumed snack foods and sugar-sweetened beverages [69]. In addition, the presence of food advertisements in games has been shown to significantly increase immediate snack and energy-dense foods consumption compared to games without food advertisements [31]. Multiple studies have consistently demonstrated that gamers have poor dietary patterns characterised by high sugar consumption and low fibre intake, with 84% failing to meet fruit and vegetable recommendations, largely due to the displacement of healthier foods by energy drinks and processed foods [48,51,53,55,100].

Additionally, gamers have been shown to have higher overall energy intake than non-gamers [30,32,42]. A previous study compared ad libitum food intake in male adolescents after sessions of video gaming or rest and found a significantly greater energy intake after gaming despite similar hunger ratings [30]. Irregular meal timing associated with video gaming was linked to higher overall caloric intake and lower diet quality in young adults, potentially reflecting compensatory eating following delayed or skipped meals [50]. Two randomised controlled trials from our group have examined the acute effects of video gaming on stress markers and food intake [32,42]. The first study demonstrated that violent video gaming significantly increased diastolic blood pressure (+7.5 mmHg) and reduced feelings of fullness compared to non-violent gaming or television [32]. The second study found that video gaming increased heart rate, blood pressure, and stress compared to television viewing, with violent gaming specifically leading to an additional 208 calories consumed, particularly from sweet foods and saturated fat [42]. The dietary patterns observed among gamers are also shaped by commercial and environmental influences. Energy drink marketing and in-game advertising may normalise frequent consumption of sugar-sweetened beverages and ultra-processed foods [53,101]. These exposures are particularly concerning for adolescents and young adults, given evidence linking energy drink consumption to cardiometabolic risk and sleep disruption [102]. The clustering of behaviours, such as late-night gaming, irregular meals, high sugar intake, and sleep deprivation, may act synergistically to increase long-term metabolic risk [103]. Therefore, interventions that address food availability, marketing exposure, and meal timing within gaming contexts may be more effective than those targeting individual behaviour.

### 3.5. Integrated Conceptual Framework and Mechanisms

**Figure 1:**
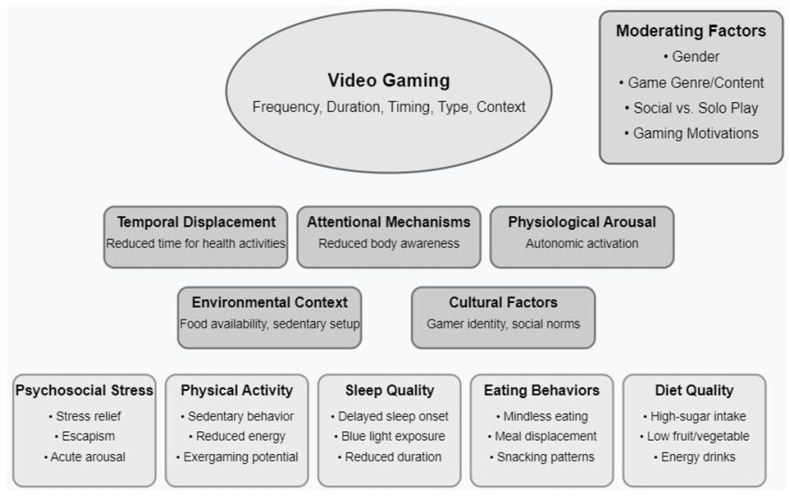
Integrated Biopsychosocial Model linking Video Gaming to Health Outcomes. Moderating factors may shape the strength or direction of these relationships.

The relationships between video gaming and health outcomes appear interconnected. An integrated bio-psychosocial framework may be useful to describe the pathways linking video gaming to health outcomes across multiple domains. This model is particularly useful as it captures the complex interactions between biological arousal, psychological immersion, and social/environmental factors that underlie gaming behaviour (Figure 1). Temporal displacement may occur as video gaming directly competes with time for health-promoting activities, including physical activity, meal preparation, and sleep [10]. Attentional mechanisms may also come into play when cognitive absorption during video gaming reduces internal resources available for sensing and monitoring physiological cues related to hunger, satiety, fatigue, and stress [29,104]. Physiological arousal may represent another mechanism, as gaming induces autonomic activation and stress responses that can disrupt sleep onset, alter appetite regulation, and influence stress perception [86,105]. The environmental context surrounding video gaming may create conditions conducive to sedentary behaviour and energy-dense food consumption [44]. These processes may operate bi-directionally and synergistically. For example, sleep disruption from evening gaming may impair subsequent executive function and self-regulation and reduce the capacity to make healthy dietary choices or engage in planned physical activity [86,88]. Similarly, stress relief obtained with video gaming may reinforce gaming behaviours despite awareness of its negative health impacts [106]. This model acknowledges significant individual variability based on video gaming context (casual vs. competitive), content (genre, game design features), timing (duration, time of day), and personal factors (sex, susceptibility to problematic use), and explains some of the heterogeneous findings found across studies. This conceptual framework may facilitate the development of testable hypotheses, for example, that sleep disruption may mediate the relationship between evening gaming and poorer dietary self-regulation, or that social gaming may moderate stress outcomes via enhanced perceived connectedness.

### 3.6. Implications and Future Directions

This review highlights several important implications for research, public health surveillance, and intervention design. Studies should prioritise longitudinal designs to disentangle directionality and identify developmental windows during which gaming may have stronger health effects. Greater attention is needed to heterogeneity in gaming exposure, including genre, social context, timing, and motivational drivers, rather than relying solely on duration-based measures. From an intervention perspective, a harm-reduction approach may be most appropriate. Rather than discouraging gaming outright, strategies could focus on optimising gaming contexts through scheduled breaks, promotion of healthier snacks, limits on late-night play, and integration of movement into gaming routines. Schools, universities, and parents may also play a role in supporting healthy gaming habits through education and environmental design. At a policy level, inclusion of gaming-specific indicators within lifestyle surveillance systems could improve monitoring of emerging health risks. Regulation of food and beverage marketing within gaming environments may also warrant consideration, particularly for younger groups.

Gaming behaviours may be screened during routine health consultations, particularly in young adults, using brief validated tools to identify individuals at risk of problematic gaming or gaming-associated lifestyle disruption. Subjects could be advised to avoid gaming in the hour before sleep, especially on mobile devices, given evidence linking evening gaming to delayed sleep onset. Scheduled breaks and structured meal times during gaming sessions are encouraged, as cognitive distraction contributes to mindless eating and increased caloric intake. Replacing energy-dense snacks and sugary beverages with healthier alternatives in the gaming environment could be recommended. A harm-reduction rather than abstinence-based approach is advised, recognising some of the psychosocial benefits of gaming. Cooperative and casual gaming may carry lower health risks than competitive or immersive long-session gaming.

A notable gap in the current evidence base is the under-representation of older adult populations. The majority of studies has focussed on younger age groups, limiting the generalisability of findings across the lifespan. This is a significant missed opportunity, as older adults may experience distinct psychosocial and physiological responses to gaming, and evidence suggests that casual and cognitive gaming may offer benefits for mental health, social connectedness, and cognitive function in this group [107,108]. Future research should prioritise longitudinal studies in older adult cohorts, examining both potential risks (e.g., sedentary behaviour, sleep fragmentation) and benefits (e.g., cognitive stimulation, reduced social isolation) of gaming in this population.

### 3.7. Strengths and Limitations

This narrative review provides a comprehensive synthesis across multiple health domains and develops an integrated bio-psychosocial framework explaining mechanisms linking gaming to health outcomes. Key strengths include a critical appraisal of the current evidence addressing some of the conflicting findings around links between video game playing and lifestyle, nutritional, and health outcomes, and identification of critical research gaps for future, more robust studies. However, important limitations need to be acknowledged. As a narrative, non-systematic review, our approach is limited and not representative of the wider evidence existing on this topic. The heterogeneous study designs, populations, gaming platforms, and measures across the reviewed literature limited definitive conclusions about associations and causal relationships. Additional limitations include reliance on self-reported gaming behaviours and dietary intake in many studies. In addition, rapid technological evolution means that findings may not generalise across gaming platforms or generations.

An additional limitation relates to the temporal relevance of some studies included in this review. The gaming landscape has rapidly changed in the last two decades as initial studies were characterised by predominantly console-based and early online gaming, prior to the widespread adoption of smartphones, live-service games, social gaming platforms, and esports. The food environments, gaming session lengths, social dynamics, and physiological exposures associated with contemporary gaming may differ substantially from those studied in this earlier period.

### 3.8. What Is Already Known on This Subject?

Previous research has established that video gaming is a prevalent leisure activity globally, with evidence suggesting associations between gaming and various health outcomes, including sleep disruption, sedentary behaviour, and altered eating patterns. Individual studies have documented specific relationships, such as the impact of gaming on physical activity levels, sleep quality, eating behaviour, and diet quality. However, the existing literature has been fragmented, focusing on isolated health domains without considering the interconnected nature of these relationships. Limited understanding existed regarding the underlying mechanisms explaining how gaming influences health behaviours, and most studies employed cross-sectional designs that precluded examination of causal pathways and temporal relationships.

### 3.9. What This Study Adds?

First, it provides an integrated multi-domain synthesis demonstrating that relationships between video gaming and health are closely connected, such as the clustering of evening gaming, sleep disruption, and poor dietary patterns, which may act synergistically to elevate metabolic risk. Second, the proposed biopsychosocial framework may generate testable hypotheses, including the mediating role of sleep disruption in the relationship between evening gaming and impaired dietary self-regulation. Third, the review provides a prioritised research agenda identifying critical gaps such as the need for more longitudinal studies, the under-representation of older adults, the need for platform, genre, and context-sensitive outcome measures beyond total gaming duration, and the need for intervention studies testing harm-reduction strategies within gaming environments.

## 4. Conclusions

Video gaming has complex health effects across the lifespan and is linked to sedentary behaviour, sleep disruption, and poor eating patterns, while also demonstrating potential health benefits. The proposed biopsychosocial model may help to explain some of these relationships and highlight significant research gaps to be addressed in future studies.

## Figures and Tables

**Table 1:** Summary of key studies investigating the association between video gaming and health outcomes across the life course.

Study	Age Group/ Population	Design	Gaming Measure	Health Domain	Key Findings	Mechanisms Identified
Reinecke et al. (2009) [24]	Adults (Mean age = 24.1 years)	Cross-sectional survey	Gaming frequency and recovery experiences	Psychosocial stress	Higher life stress predicted greater gaming for recovery; Gaming provided psychological detachment from stressors	Recovery experiences; Psychological detachment; Relaxation
Ballard et al. (2009) [25]	Young adult males	Cross-sectional survey	Video game screen time	Physical activity; BMI; Other media use	Frequent gamers reported significantly less physical activity; Gaming time predicted BMI independent of other media use	Time displacement; Sedentary behaviour
Weaver et al. (2009) [26]	Adults; *n* = 562	Cross-sectional survey	Gaming status (player vs. non-player)	BMI; Mental health; Physical health	Female gamers reported greater depression and poorer health; Male gamers had higher BMI; Both sexes showed greater reliance on internet-based social support	Sedentary behaviour; Sex-specific health risk profiles
Weaver et al. (2010) [27]	Adolescent males (mean age = 16.6 years); *n* = 13	Randomised crossover experimental study	1 h pre-sleep gaming vs. DVD watching	Sleep onset latency; Sleepiness; Sleep architecture	Pre-sleep gaming increased sleep onset latency and reduced subjective sleepiness; Sleep architecture was unaffected	Cognitive arousal; Evening alertness
Oldham-Cooper et al. (2011) [28]	Young adults	Controlled experimental study	Computer game playing during lunch vs. focused eating	Satiety; Meal memory; Later snacking	Gaming during meals led to reduced fullness ratings, poorer meal recall, and increased later snacking	Attentional allocation; Mindless eating; Memory encoding disruption
Snodgrass et al. (2011) [29]	Adults (18–30 years)	Mixed methods: Interviews + web survey	Immersive online gaming (World of Warcraft)	Psychosocial stress	Over 50% reported gaming improved mood and reduced stress; Deep immersion facilitated “dissociation” from stressors	Escapism; Attentional redirection; Psychological absorption
Chaput et al. (2011) [30]	Adolescent males (15–19 years)	Randomised crossover design	1 h video game session vs. rest period	Ad libitum food intake; Physiological measures	Significantly higher caloric intake after gaming despite similar hunger ratings; Positive energy balance	Stress-induced eating; Cognitive stimulation; Reward processing
Cronin & McCarthy (2011) [31]	Young adults (18–30 years)	Ethnographic exploration	Gaming identity and behaviour	Food culture; Eating practices	Identified a distinct “gaming food culture” valuing convenience, minimal preparation, and energy-dense options	Subcultural identity; Value system around food; Social norms
Siervo et al. (2013) [32]	Young men (Mean age = 23.1 years)	Randomised controlled trial (three-arm)	1 h of violent video game vs. non-violent video game vs. TV watching	Blood pressure; Appetite perception; Food preferences	Violent videogame playing significantly increased diastolic BP (+7.5 ± 5.8 mm Hg); Players of violent games felt less full and reported a preference for sweet foods	Physiological stress response; Arousal-induced appetite changes; Game content-specific effects
Nishiwaki et al. (2014) [33]	Young adults (mean age = 31 years); *n* = 20	12-week randomised crossover intervention	Gamified activity monitor vs. standard monitor	Daily steps; Physical activity; Body composition	Gamified intervention produced significantly more daily steps, greater physical activity intensity, and greater body fat reduction compared to standard monitoring	Gamification of physical activity; Motivational engagement
Mario et al. (2014) [22]	Young men (18–24 years)	Cross-sectional comparison	Frequent vs. non-frequent gaming	Central adiposity; Dietary intake	Frequent gamers showed significantly higher sugar consumption, lower fibre intake, and greater central adiposity	Dietary displacement; Food environment; Energy-dense snacking
Exelmans & Van den Bulck (2015) [34]	Population-based sample (18–94 years)	Cross-sectional survey	Gaming volume, timing, and content	Sleep quality; Chronotype	Gaming volume is significantly associated with later bedtimes, longer sleep onset latency, and greater daytime fatigue	Evening arousal; Delayed sleep phase; Blue light exposure
Simons et al. (2015) [35]	Adolescents (aged 12–16 years)	24 h recall diary study	Active vs. non-active gaming time	Physical activity; Snack consumption	Active gaming did not displace sedentary gaming or other physical activities; active gaming time was weakly associated with increased snack consumption	Limited net energy balance benefit; Snack association
Harbard et al. (2016) [36]	Young adults (18–35 years)	14-day daily diary study	Evening gaming (type, duration, timing)	Sleep parameters (diary and actigraphy)	Each hour of gaming after 8 pm is associated with 28 min delay in sleep onset; Stronger effect than evening studying	Circadian phase delay; Evening arousal; Blue light exposure
Howe et al. (2016) [37]	Young adults (aged 18–35 years); *n* = 1182	Cohort study	Pokémon GO installation and playing status	Physical activity (daily step count)	Pokémon GO was associated with a short-term increase in daily steps; however, the effect attenuated progressively and returned to pre-installation levels by six weeks	Augmented reality gaming; Incidental physical activity; Novelty-driven motivation; Transient behaviour change
Smith et al. (2017) [38]	Young adults (18–25 years)	Cross-sectional survey	Multiplayer vs. single-player gaming preferences	Sleep timing; Duration	Multiplayer online games associated with later bedtimes compared to single-player games	Social obligation; Reduced autonomy over session duration; Gaming communities
Turel et al. (2017) [39]	Children/adolescents (mean age = 13.1 years); *n* = 125	Cross-sectional time-lagged cohort	Pre-bedtime gaming duration; Session duration	Abdominal adiposity; Sleep quality; Sweet drink consumption	Pre-bedtime gaming was associated with greater abdominal adiposity, mediated through poor sleep quality and higher sweet drink consumption	Sleep disruption; Sugar-sweetened beverage consumption; Mediated pathways to obesity
Cha et al. (2018) [40]	Adolescents (13–19 years)	Cross-sectional survey	Gaming sessions > 6 h	Eating behaviours; BMI	Long gaming sessions are associated with meal skipping, late-night eating, and increased BMI	Temporal displacement; Irregular eating patterns
Zurita-Ortega et al. (2018) [41]	Young adults	8-week intervention	Active video games intervention	Physical fitness; Body composition	Significant improvements in physical fitness measures following active gaming intervention compared to controls	Physical exertion; Motivational engagement; Gamification of exercise
Siervo et al. (2018) [42]	Young men (18–30 years)	Randomised controlled crossover trial	1 h standardised gaming session vs. television viewing	Stress biomarkers; Eating behaviour	Gaming produced higher cortisol and blood pressure responses than TV; Higher energy intake following gaming sessions	Physiological arousal; Stress-induced eating; Attentional mechanisms
Altintas et al. (2019) [43]	Young adults (mean age = 24.4 years); *n* = 217	Cross-sectional survey	Weekly gaming duration; Gaming intensity	Sleep quality (PSQI)	Nearly 40% of gamers had poor sleep quality; gaming intensity was a stronger predictor of poor sleep than duration	Physiological arousal; Cognitive alertness
Puolitaival et al. (2020) [44]	Adolescent males (mean age = 17.8 years); *n* = 796	Cross-sectional population-based survey	Gaming >3 h/day vs. ≤3 h/day	Physical activity; Dietary habits; BMI	Heavy gamers had lower physical activity, lower fruit/vegetable intake, higher sweetened drink consumption, and greater sitting time	Temporal displacement; Sedentary behaviour; Dietary displacement
Potvin Kent et al. (2019) [45]	Content analysis of 100 popular video games	Content analysis	N/A—examined games, not players	Food marketing	84% of advertised food products failed nutritional quality standards; Energy drinks most common product category	Marketing exposure; Brand association; Cultural influence
Koban et al. (2022) [46]	Young adults	Longitudinal (semester-long)	Gaming frequency, compensatory gaming motivation	Psychosocial stress; Academic performance	Gaming for escape during exam periods predicted poorer stress management and academic outcomes	Maladaptive coping; behavioural avoidance; Reduced problem-solving
Akcay & Akcay (2020) [47]	Young adults	Cross-sectional survey	Computer game playing habits (frequency, duration, timing)	Sleep quality (Pittsburgh Sleep Quality Index)	Heavy gamers (>3 h/day) scored significantly worse on sleep quality compared to moderate/non-gamers	Sleep latency; Sleep efficiency; Bedtime displacement
Rudolf et al. (2020) [48]	Adults (18+ years)	Online survey (eSports study)	Competitive vs. recreational gaming	Dietary intake; Physical activity	84% failed to meet “five a day” fruit/vegetable recommendations; Competitive gamers showed higher energy drink consumption	Performance enhancement seeking; Gaming culture norms
Kwok et al. (2021) [49]	Young adults	Cross-sectional survey	Gaming frequency and duration	Physical activity; Sleep quality; Academic performance	Excessive gaming (>2 h/day) is negatively associated with exercise levels and sleep quality	Temporal displacement; Sleep disruption; Sedentary behaviour
Vaarala et al. (2022) [50]	Adolescents/young adults (15–21 years)	Cross-sectional survey	Problematic Gaming Inventory	Eating behaviours; Food attitudes	Problematic gamers reported higher rates of distracted eating, convenience food preferences, and barriers to healthy eating	Attentional mechanisms; Food environment; Cooking skill barriers
Moore & Morrell (2024) [51]	College men (aged 18–24 years); *n* = 1259	Cross-sectional study	Non-, moderate, and high gaming groups	Dietary patterns (3-day food records)	High gamers had greater saturated fat and discretionary calorie intake and lower fruit and vegetable consumption compared to non-gamers	Food accessibility; Gaming subculture; Dietary displacement
Matias et al. (2023) [52]	Adults (18–35 years)	Cross-sectional study	Gaming patterns (frequency, duration, genre)	Mental health; Physical activity; Eating habits; Sleep patterns	High gameplay time (>20 h/week) associated with lower sleep quality and physical activity; Gaming genre influenced sleep timing	Digital immersion; Variable reinforcement scheduling; Temporal displacement
Soffner et al. (2023) [53]	Adults (mean age = 24.2 years); *n* = 817	Cross-sectional survey	Weekly gaming duration	Dietary intake; Fluid intake	Gaming time positively correlated with energy drink, soft drink, and fast food consumption; Fruit and vegetable intake was low	Gaming culture norms; Convenience prioritisation
Kaewpradup et al. (2025) [54]	Young adults	Cross-sectional survey	Gaming patterns (frequency, duration, genre)	Dietary intake; Physical activity; Sleep quality; Psychosocial Stress	High gameplay time (>10 h/week) is associated with lower sleep quality, greater BMI, and lower dietary quality	Convenience prioritisation; Temporal displacement; Sleep disruption
Caycho et al., (2025) [55]	Young adults	Cross-sectional survey	Gaming patterns (frequency, duration)	Dietary intake; Physical activity;	Peer interaction within the gaming environment and the perceived influence of video games were significantly associated with poorer eating habits.	Digital immersion; Temporal displacement
Giller et al. (2025) [14]	Adults (mean age = 27 years); *n* = 243	Cross-sectional survey	Pokémon GO playing habits	Physical activity; Mental well-being; Sleep; Social interaction	Pokémon GO was associated with higher physical activity and improved mood; however, a notable proportion of players reported sleep sacrifice, addictive use, and exceeding WHO screen time guidelines	Augmented reality gaming; Incidental physical activity; Addictive potential; sleep displacement

## Data Availability

No new data were created or analyzed in this study.
